# Key aroma contributors and perceptual interactions of dried longan-like aroma in lapsang souchong black tea with different natural aging times

**DOI:** 10.1016/j.fochx.2026.104445

**Published:** 2026-09-11

**Authors:** Yutao Shi, Lirong Zuo, Canyang Huang, Yanhua Zhou, Xi Cheng, Yan Huang, Xiaoyong Wang, Yucheng Zheng, Feiquan Wang, Bo Zhang, Shulin Zheng

**Affiliations:** a College of Tea and Food Sciences, Wuyi University, Wuyishan 354300, Fujian, China; b Tea Engineering Research Center of Fujian Higher Education, Wuyi University, Wuyishan 354300, Fujian, China; c Department of Management, Minbei Vocational and Technical College, Yanping, 354200, Fujian, China; d College of Mechanical and Electrical Engineering, Huangshan University, Huangshan 245021, Anhui, China

**Keywords:** Lapsang souchong black tea, Volatile aroma compounds, Dried longan-like aroma, Perceptual interaction, Molecular simulation

## Abstract

This study investigated aroma evolution in Lapsang Souchong black tea (LS) with different natural aging times and the perceptual basis of its dried longan-like aroma. Sensory evaluation, HS-SPME-GC–MS, aroma recombination/omission, S-curve–Feller analysis, and molecular simulations showed that, with increasing natural aging time, LS aroma shifted from pine-smoke, sweet, and fruity aroma dominance to prominent dried longan-like aroma, followed by enhanced woody and aged aromas. LS19 showed the highest dried longan-like aroma intensity. Benzaldehyde, Geranyl acetate, and 2-Acetylfuran were identified as key contributors. Binary mixtures mainly showed additive effects, whereas the ternary system exhibited a synergistic effect. Molecular simulations suggested stable binding of the key aroma compounds with the candidate olfactory receptor OR1A1 (−53.89 kcal/mol), with ILE126 and VAL227 identified as key binding residues. These findings clarify the material basis and perceptual features of dried longan-like aroma and support LS quality evaluation, storage management, and aroma regulation.

## Highlights

• The strongest dried longan-like aroma was found in 4-year-aged LS.
• Benzaldehyde, geranyl acetate, and 2-acetylfuran were key contributors.
• Dried longan-like aroma was synergistically enhanced in the ternary system.
• ILE126 and VAL227 were identified as key residues stabilizing OR1A1 binding.

## Graphical abstract

**Figure:**
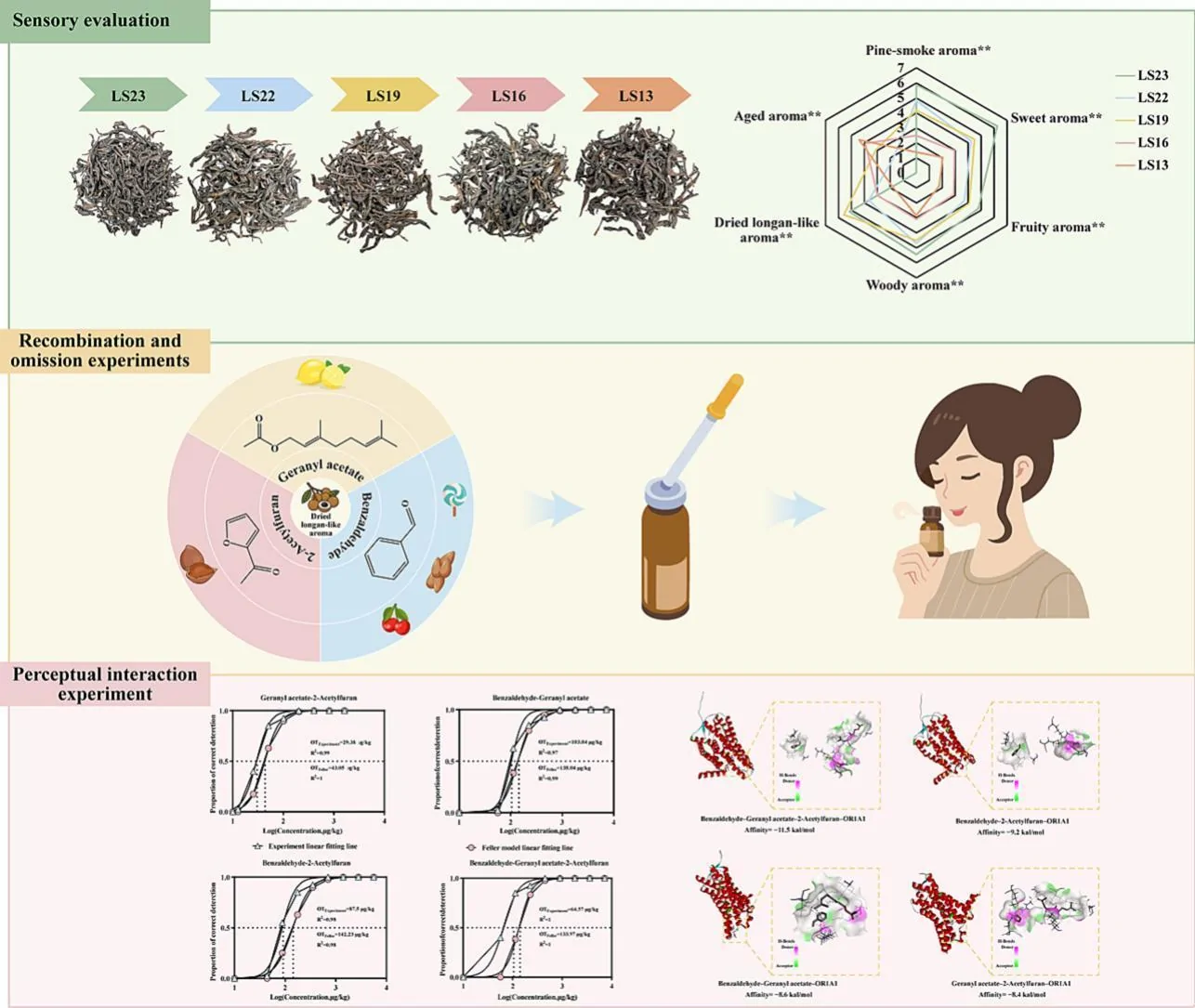

## 1 Introduction

Lapsang Souchong black tea (LS) is one of the representative traditional black teas in China. It originated from the Tongmu region of Wuyi Mountain, Fujian Province, and is highly recognizable because of its distinctive pinewood-smoking process and characteristic aroma profile (Han et al., 2021). Traditional LS processing generally includes withering, rolling, fermentation, drying, and pinewood smoking. During these processes, oxidative transformation of tea constituents is promoted by fermentation, while the formation, transformation, and enrichment of volatile compounds are jointly affected by smoking and drying (Su et al., 2024). Previous studies have shown that LS is characterized by several typical aroma attributes, including pine-smoke aroma, sweet aroma, floral-fruity aroma, woody aroma, and dried longan-like aroma. Among these attributes, dried longan-like aroma, which is associated with sweet, dried-fruit-like, caramel-like, and mild fruity notes, is regarded as an important sensory characteristic of LS aroma (Yang & Chiang, 2019). In addition to processing, storage is also an important factor affecting tea aroma quality. During storage, volatile compounds may undergo oxidation, degradation, volatilization, precursor transformation, and interactions among components, leading to changes in the aroma profile of tea (P. Zheng et al., 2025). However, current studies on LS aroma have mainly focused on volatile composition, the influence of smoking processes, and overall aroma characteristics. The changes in aroma characteristics and the perceptual basis of dried longan-like aroma in LS with different natural aging times remain insufficiently understood.

Food aroma is generally composed of multiple volatile compounds, and its overall perception is not a simple linear superposition of individual odorants. Instead, it is jointly influenced by compound concentration, odor threshold, odor similarity, and interactions among components (Ye et al., 2023). Additive, synergistic, or masking effects may occur among aroma compounds, allowing aroma mixtures to exhibit sensory characteristics that differ from those of individual components (Zheng et al., 2025). Therefore, GC–MS identification, rOAV calculation, or correlation analysis alone is insufficient to fully elucidate the perceptual basis of complex aroma attributes. Aroma recombination and omission experiments can be used to verify the actual sensory contributions of candidate aroma compounds, while S-curve analysis combined with the Feller additive model provides a quantitative approach for characterizing perceptual interactions among aroma components, including additive, synergistic, and masking effects (Cometto-Muñiz et al., 2002; Niu et al., 2020; Tian et al., 2024). In addition, molecular docking and molecular dynamics simulation can provide hypothetical explanations for the potential recognition of key aroma compounds from the perspective of olfactory receptor binding and conformational stability (Tong et al., 2025). Nevertheless, the perceptual interactions among key compounds contributing to dried longan-like aroma in LS, as well as their potential binding characteristics with olfactory receptors, have not yet been systematically investigated.

Based on these considerations, LS samples with different natural aging times were used in this study. Sensory evaluation, HS-SPME-GC–MS, aroma recombination and omission experiments, S-curve–Feller additive model analysis, and molecular simulation were integrated to systematically investigate the changes in aroma characteristics, the key contributors to dried longan-like aroma, and its complex perceptual basis in LS with different natural aging times. The objectives of this study were: (1) to compare the aroma characteristics and volatile composition of LS with different natural aging times and clarify the aging-related variation of dried longan-like aroma; (2) to screen candidate aroma compounds closely associated with dried longan-like aroma and verify their actual sensory contributions through aroma recombination and omission experiments; and (3) to characterize the perceptual interactions among key contributors and explore their potential binding characteristics with the candidate olfactory receptors OR1A1 and OR2W3 using molecular docking, followed by molecular dynamics simulation of the OR1A1–ligand complexes. This study is expected to provide new insight into the natural aging-related evolution of LS aroma characteristics and the material basis and perceptual features of dried longan-like aroma, thereby offering a candidate chemical basis and theoretical reference for LS aroma quality evaluation and targeted regulation of characteristic aroma.

## 2 Materials and methods

### 2.1 Tea samples

All LS tea samples were obtained from Wuyishan Hefeng Jiaye Tea Factory (Wuyishan, Fujian, China). Fresh leaves used for both the freshly processed and aged samples were harvested from the same tea garden located in Tongmu Village, Xingcun Town, Wuyishan City, Fujian Province, China. The raw material consisted of fresh leaves of Wuyi Cai Cha, plucked according to the standard of one bud with two or three leaves. Wuyi Cai Cha is a local landrace population of *Camellia sinensis* (L.) O. Kuntze var. *sinensis* cv. ‘Bohea’ native to the Wuyi Mountain region.

The LS samples were prepared following the traditional procedure described in DB35/T 1228—2015. The traditional manufacturing process of LS generally comprises withering, rolling, fermentation, drying, and pinewood smoking (Su et al., 2024). Briefly, fresh leaves (one bud with two or three leaves) were plucked in May and immediately transported to the processing factory. The leaves were first withered under sunlight for 6 h, followed by further withering over a pinewood fire at 40 °C for 2.5 h. The withered leaves were rolled into strips at room temperature (approximately 25 °C), placed in bamboo containers, and fermented under warm and humid conditions for 8 h. Finally, the fermented leaves were subjected to pinewood smoke drying. Specifically, leaves were evenly spread on bamboo sieves in a drying room, where pinewood smoke ascended from the bottom and passed upward through the leaves at approximately 90 °C for 12 h.

The aged samples were produced during the spring seasons of 2013, 2016, 2019, 2022, and 2023. All samples were manufactured from fresh leaves harvested from the same tea garden using the identical traditional procedure described above. After processing, all samples were sealed in aluminum foil bags and naturally stored in the same warehouse. The warehouse was kept dry and well-ventilated, with indoor temperature maintained at ≤30 °C and relative humidity at ≤50%. All samples were naturally aged under these conditions with consistent storage management throughout the aging period. Three independent batches collected from different production lots within the same year were used as biological replicates for each aging group. All samples were subsequently stored at −20 °C until analysis. At the time of sampling in 2023, LS13, LS16, LS19, LS22, and LS23 had been naturally aged for 10, 7, 4, 1, and 0 years, respectively, with LS23 representing the newly processed sample of the current year.

### 2.2 Sensory evaluation and quantitative descriptive analysis

Sensory evaluation of the aroma of LS samples with different natural aging times was performed according to GB/T 23776-2018 (Liao et al., 2026). Briefly, 3.0 g of tea sample was accurately weighed into a standard sensory evaluation cup, infused with 150 mL of boiling water, and steeped for 5 min. Immediately after infusion, the aroma of the tea infusion was evaluated by the panelists.

Quantitative descriptive analysis (QDA) was further performed to evaluate six aroma attributes, namely pine-smoke aroma, sweet aroma, fruity aroma, woody aroma, dried longan-like aroma, and aged aroma. A 7-point intensity scale was used, where 0 indicated the absence of the aroma attribute, 4 indicated moderate intensity, and 7 indicated the highest intensity (Zhang et al., 2025). Each sample was evaluated in three biological replicates. The sensory panel consisted of 15 experienced professional assessors, including seven males and eight females, aged 25–60 years, all from Wuyi University. The panelists had more than 10 years of sensory evaluation experience on average. According to the international sensory analysis standard ISO 8586:2012, the assessors were screened and trained, and exercises on odor stimulus recognition and aroma intensity scoring were conducted. Standard reference materials were repeatedly sniffed by the assessors to ensure a consistent understanding of the definitions and intensity scales of each aroma attribute (**Supplementary Table S1**). All panelists provided informed consent before participating in the sensory evaluation and QDA experiments. They confirmed that they voluntarily participated in the study, fully understood the experimental procedures, and were informed of their right to withdraw from the study at any time.

### 2.3 Analysis of aroma compounds by HS-SPME-GC–MS

The aroma compounds in five LS samples with different natural aging times, namely LS13, LS16, LS19, LS22, and LS23, were analyzed using HS-SPME-GC–MS.

The HS-SPME conditions were as follows. Before extraction, a 120 μm DVB/CWR/PDMS fiber was conditioned in a Fiber Conditioning Station at 250 °C for 5 min. Tea powder, 1.0 g, was accurately weighed into a headspace vial, followed by the addition of 3 mL of saturated NaCl solution and 20 μL of methyl 2-biphenylcarboxylate internal standard solution at a concentration of 10 μg/mL. The vial was sealed and equilibrated with shaking at 100 °C for 5 min. The SPME fiber was then inserted into the headspace of the vial and exposed at the same temperature for 15 min. After extraction, the fiber was immediately inserted into the GC injection port and desorbed at 250 °C for 5 min for GC–MS separation and detection.

The gas chromatographic conditions were as follows. Volatile compounds were separated using a DB-5MS capillary column (30 m × 0.25 mm × 0.25 μm; Agilent J&W Scientific, Folsom, CA, USA). High-purity helium (>99.999%) was used as the carrier gas at a constant flow rate of 1.2 mL/min. The inlet temperature was set at 250 °C, and injection was performed in splitless mode. The solvent delay time was 3.5 min. The oven temperature program was as follows: the initial temperature was maintained at 40 °C for 3.5 min, increased to 100 °C at 10 °C/min, further increased to 180 °C at 7 °C/min, and finally increased to 280 °C at 10 °C/min and held for 5 min.

The mass spectrometric conditions were as follows. Electron ionization (EI) was used at an electron energy of 70 eV. The ion source, quadrupole, and transfer line temperatures were set at 230, 150, and 280 °C, respectively. Mass spectra were acquired in full-scan mode over an *m*/*z* range of 50–500. Aroma compounds were identified based on retention indices (RIs) and mass spectral matching. RIs were calculated using a C7–C40 n-alkane series analyzed under the same chromatographic conditions, and the results were compared with those in the NIST database for qualitative identification. Each sample was analyzed in triplicate. Semi-quantitative analysis of aroma compounds was performed using the internal standard method according to the following equation:

$${\mathrm{RC}}_{\mathrm{voc}}=\frac{A_{\mathrm{voc}}}{A_{\mathrm{std}}}\times \frac{m_{\mathrm{std}}}{m_{s}}$$

where *RC*_*voc*_ represents the relative content of the aroma compound, expressed as μg/kg; *A*_*voc*_ is the chromatographic peak area of the aroma compound; *A*_*std*_ is the peak area of the internal standard; *m*_*std*_ is the mass of the internal standard added to the sample, expressed as μg; and *m*_*s*_ is the mass of the tea sample, expressed as g.

### 2.4 Calculation of rOAV

Relative odor activity value (rOAV) was used to evaluate the contribution of aroma compounds to the overall aroma of the samples. It was calculated by dividing the estimated concentration of each volatile organic compound by its odor threshold in water (Huang et al., 2026). The rOAV was calculated as follows:

$$\mathrm{rOAV}=\frac{{\mathrm{RC}}_{\mathrm{voc}}}{{\mathrm{OT}}_{i}}$$

where *RC*_*voc*_ represents the relative content of the compound, expressed as μg/kg, and *OT*_*i*_ represents the odor threshold of the aroma compound, expressed as μg/kg.

### 2.5 Aroma recombination and omission experiments

The deodorized tea powder matrix was prepared according to the method of Du et al. (2013), with minor modifications. Briefly, ground samples of LS13, LS16, LS19, LS22, and LS23 were mixed in equal proportions and transferred into a 1-L round-bottom flask, followed by the addition of 300 mL of ultrapure water. The mixture was subjected to rotary evaporation at 40 °C. Ultrapure water was replenished as needed during rotary evaporation, and the aroma of the tea powder was periodically evaluated. The treatment was continued until no obvious aroma could be perceived. The deodorized tea powder was subsequently dried at 60 °C for 3 h and stored in sealed containers until use.

To verify the deodorization efficiency, the deodorized tea powder matrix was analyzed by HS-SPME-GC–MS using the same analytical conditions described in Section 2.3. The total ion chromatogram showed low overall volatile signal intensity in the deodorized tea powder matrix **(Supplementary Fig. S1)**. In addition, Benzaldehyde, Heptanal, 2-Methylphenol, Geranyl acetate, 2-Acetylfuran, and Eugenol were specifically examined, and none of these six candidate aroma compounds was detected in the deodorized tea powder matrix, indicating that the matrix had a low aroma background and was suitable for subsequent aroma recombination and omission experiments.

Aroma recombination experiments were conducted to verify the contribution of candidate aroma-active compounds to dried longan-like aroma in LS. Benzaldehyde, Heptanal, 2-Methylphenol, Geranyl acetate, 2-Acetylfuran, and Eugenol were selected as candidate aroma compounds. Referring to the method of Chen et al. (2025), LS19, which showed the highest intensity of dried longan-like aroma, was selected as the representative sample. The selected compounds were added to the deodorized tea powder matrix according to their measured concentrations in LS19 to construct the complete aroma recombination model. All model samples were infused under the same conditions described in Section 2.2, namely 3.0 g of sample with 150 mL of boiling water for 5 min before evaluation. The aroma recombination experiment was performed by the same sensory panel described in Section 2.2. Before the experiment, the assessors received additional training in the recognition and intensity scoring of dried longan-like aroma. A triangle test was used to compare the overall aroma difference between the original LS19 sample and the complete recombination model, and the intensity of dried longan-like aroma was evaluated using the 7-point scale.

For the omission experiment, the complete recombination model was used as the control. Single-omission models were prepared by individually removing Benzaldehyde, Heptanal, 2-Methylphenol, Geranyl acetate, 2-Acetylfuran, or Eugenol from the complete model. Triangle tests were used to compare each omission model with the complete recombination model in terms of overall aroma difference. In addition, QDA scoring was performed to evaluate changes in the intensity of dried longan-like aroma. A compound was considered a key contributor to dried longan-like aroma in LS when its omission resulted in a significant sensory difference from the complete recombination model and a significant decrease in dried longan-like aroma intensity. All recombination and omission models were prepared using the same batch of deodorized tea powder and infused under identical conditions to ensure that potential matrix-dependent adsorption and release effects were controlled consistently across the compared models. All samples were coded with random three-digit numbers and presented in randomized order. Each test was repeated three times. The significance of the triangle test results was determined using the binomial distribution.

### 2.6 S-curve analysis

To characterize the interactions among Benzaldehyde, Geranyl acetate, and 2-Acetylfuran in the perception of dried longan-like aroma, S-curve analysis combined with the Feller additive model was applied to evaluate the perceptual interaction types of binary and ternary mixture systems. The Feller additive model is based on the response-addition principle under the assumption of independent detection of individual odorants and has been widely applied to characterize perceptual interactions among aroma compounds, including additive, synergistic, and masking effects (Cometto-Muñiz et al., 2002; Niu et al., 2020; Tian et al., 2024). The odor thresholds reported in the literature for the three compounds were used as reference concentrations, and eight concentration levels were prepared at 16, 8, 4, 2, 1, 0.5, 0.25, and 0.125 times the corresponding threshold values (Yuan et al., 2024). In the mixture systems, each component was prepared at the same threshold multiple. Three binary systems, namely Benzaldehyde–Geranyl acetate, Benzaldehyde–2-Acetylfuran, and Geranyl acetate–2-Acetylfuran, and one ternary system, Benzaldehyde–Geranyl acetate–2-Acetylfuran, were constructed. All systems were prepared using deodorized tea powder as the matrix and infused under the conditions described in Section 2.2 before odor threshold determination.

Odor thresholds were determined using the three-alternative forced-choice (3-AFC) method. In each test, three randomly coded samples were presented to each assessor, including one sample containing the target aroma compound or mixture and two blank matrix samples. The assessors were asked to identify the different samples, and the raw detection rate at each concentration level was calculated accordingly. To eliminate the influence of random guessing, the detection probability was corrected using the following equation:

$$P=\frac{P_{\mathrm{raw}}-1/3}{1-1/3}$$

where *P* represents the corrected detection probability and *P*_raw_ represents the raw detection probability. When *P* < 0, the value was set to 0. The logarithm of the concentration was then used as the x-axis, and the corrected detection probability was used as the y-axis. The data were fitted using the following sigmoid function:

$$P=\frac{1}{1+\exp\left[-\left(x-x_{0}\right)/b\right]}$$

where *x* is the logarithmic concentration of the aroma compound or mixture [log(μg/kg)], *x*_*0*_ is the logarithmic concentration corresponding to the odor threshold, and b is the slope parameter of the curve. The concentration corresponding to *P* = 0.5 was defined as the experimental threshold. The concentration of each mixture system was expressed as the sum of the concentrations of all components in the system.

The theoretical thresholds of the mixture systems were calculated based on the Feller additive model. The theoretical detection probabilities of the binary and ternary systems were calculated using the following equations:

$$P_{\mathrm{AB}}=P_{A}+P_{B}-P_{A}P_{B}$$

$$P_{\mathrm{ABC}}=1-\left(1-P_{A}\right)\left(1-P_{B}\right)\left(1-P_{C}\right)$$

where *P*_A_, *P*_B_, and *P*_C_ represent the corrected detection probabilities of the individual components at the corresponding concentrations. The theoretical S-curves were then obtained according to the Feller additive model, and the concentration corresponding to *P* = 0.5 was defined as the theoretical threshold. The threshold ratio *Y* was further calculated as follows:

$$Y=\frac{T_{\exp}}{T_{\mathrm{theo}}}$$

where *T*_*exp*_ is the experimentally determined threshold of the mixture system, and *T*_*theo*_ is the theoretical threshold calculated using the Feller additive model. The perceptual interaction type was classified according to the *Y* value: *Y* > 1 indicated a masking effect; *Y* = 1 indicated no apparent interaction; 0.5 < *Y* < 1 indicated an additive effect; and *Y* < 0.5 indicated a synergistic effect.

### 2.7 Molecular docking

The SDF-format ligand files of Benzaldehyde, Geranyl acetate, and 2-Acetylfuran were downloaded from the PubChem database (https://pubchem.ncbi.nlm.nih.gov/). The three-dimensional structure models of OR1A1 and OR2W3 were obtained from the AlphaFold Protein Structure Database. Molecular docking was performed using AutoDock Vina 1.2.5, with the exhaustiveness parameter set to 8. The docking results were evaluated based on binding affinity, expressed as kcal/mol, and conformations with a root-mean-square deviation (RMSD) of less than 2 Å were regarded as valid. The docking results were further analyzed and visualized using Discovery Studio 2019.

### 2.8 Molecular dynamics simulation

Molecular dynamics (MD) simulations were performed using GROMACS 2025.1 for four complexes: Benzaldehyde–Geranyl acetate–2-Acetylfuran–OR1A1, Benzaldehyde–2-Acetylfuran–OR1A1, Benzaldehyde–Geranyl acetate–OR1A1, and Geranyl acetate–2-Acetylfuran–OR1A1. The AMBER99SB force field and SPC water model were used. The time step was set to 2 fs, and long-range electrostatic interactions were treated using the particle mesh Ewald (PME) method. The systems were first subjected to energy minimization, followed by equilibration under the NVT ensemble for 100 ps and under the NPT ensemble for 100 ps. The system temperature was maintained at 300 K, the total simulation time was 100 ns, and the cutoff distance was set to 1.2 nm. System stability and convergence were evaluated by monitoring root-mean-square deviation (RMSD), root-mean-square fluctuation (RMSF), and potential energy. Binding free energy calculations and alanine scanning were performed using the gmx_MMPBSA program, and the results were visualized using GraphPad Prism 9. In addition, free energy landscapes were constructed and analyzed based on RMSD, radius of gyration (Rg), and Gibbs free energy, and the corresponding plots were generated using Origin 2024.

### 2.9 Statistical analysis

All measurements were performed in triplicate, and data are presented as mean ± standard deviation (*n* = 3). One-way analysis of variance (ANOVA) followed by Tukey's multiple comparison test was conducted using IBM SPSS Statistics 26.0 (IBM Corp., Armonk, NY, USA); differences with *P* < 0.05 were considered statistically significant. Multivariate analysis of aroma data was performed on the MetaboAnalyst 6.0 platform (https://www.metaboanalyst.ca/). Radar charts were plotted in Microsoft Excel 2019 (Microsoft Corp., Redmond, WA, USA), and box plots, stacked plots, and S-curve plots were generated using GraphPad Prism 9.0 (GraphPad Software, San Diego, CA, USA).

## 3 Results and discussion

### 3.1 Sensory characteristics

**Fig. 1:**
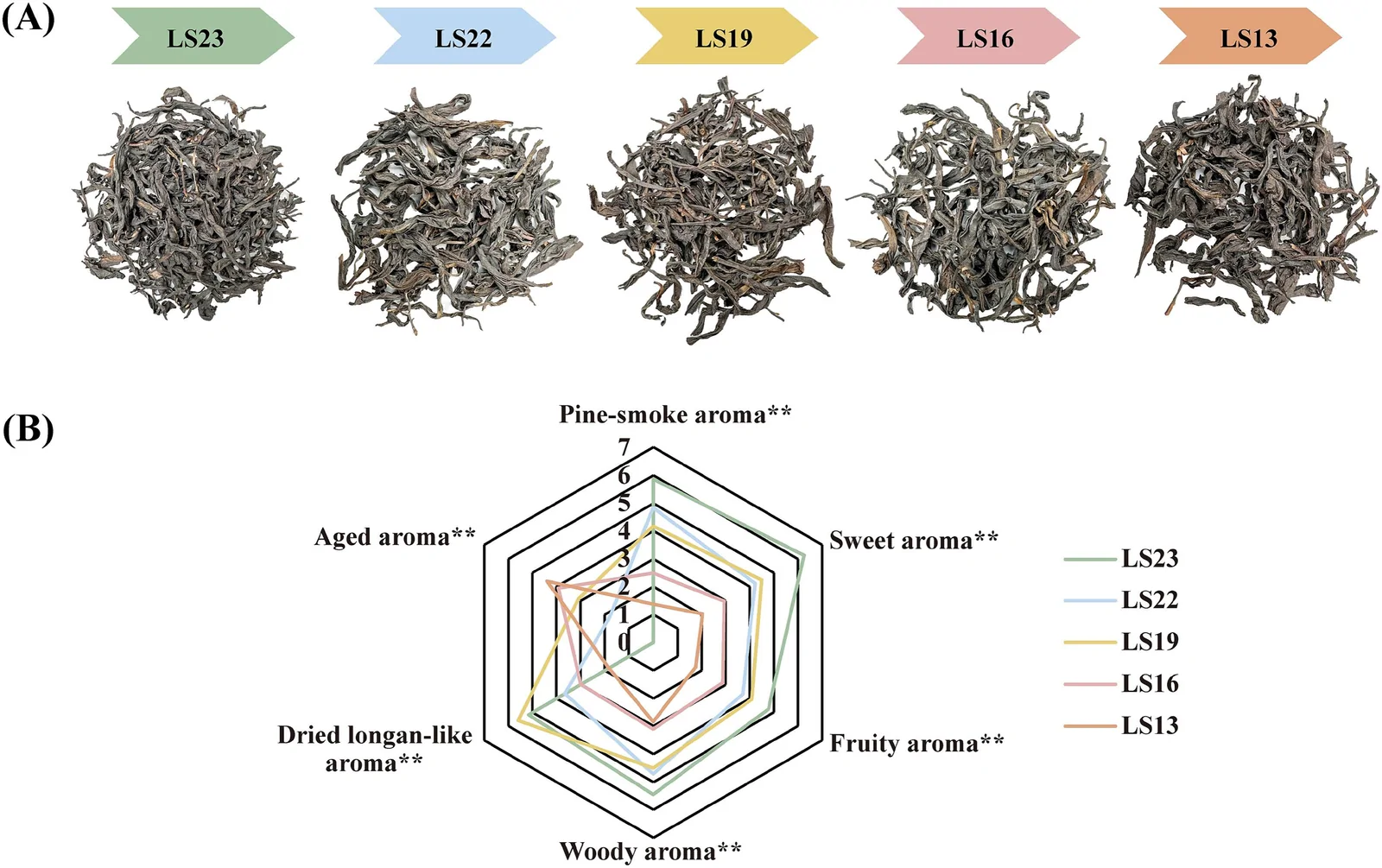
Sensory characteristics of LS from different years. (A) Dry tea appearance of LS from different years. (B) Radar plot of aroma attributes of LS from different years. Significant differences are indicated by *P < 0.05 and **P < 0.01.

As shown in Fig. 1A, the dry tea leaves of LS samples with different natural aging times showed generally similar appearances. No obvious changes were observed in leaf shape or color among the samples with increasing natural aging duration. All samples exhibited tightly twisted and robust strips with a dark and glossy appearance, indicating that the typical dry tea quality characteristics of LS were retained. To systematically characterize the aroma profiles of LS samples with different natural aging times, sensory evaluation combined with QDA was performed. The results showed that, with increasing natural aging time, the aroma profile of LS gradually shifted from being dominated by pine-smoke aroma, sweet aroma, and fruity aroma to a profile characterized by prominent dried longan-like aroma, and subsequently by enhanced woody aroma and aged aroma **(**Fig. 1B**)**. Specifically, LS23 was mainly characterized by pine-smoke aroma, sweet aroma, and fruity aroma, with intensities of 5.87, 6.25, and 4.77, respectively, indicating that the newly processed sample retained strong smoky, sweet, and fruity notes. Compared with LS23, the intensities of pine-smoke aroma and sweet aroma in LS22 decreased to 4.84 and 4.22, respectively, whereas woody aroma remained relatively strong, with an intensity of 4.71. This result suggested that the aroma profile of LS22 shifted from a pine-smoke aroma- and sweet aroma-dominant profile toward a profile characterized by pine-smoke aroma and woody aroma. Among all samples, LS19 exhibited the highest intensity of dried longan-like aroma, reaching 5.59. Moderate intensities of sweet aroma, fruity aroma, and pine-smoke aroma were also observed in LS19, with scores of 4.49, 4.08, and 4.18, respectively. These results indicated that LS19 was characterized by the coexistence of dried longan-like aroma with sweet, fruity, and pine-smoke notes. From LS19 to LS16 and LS13, the intensities of dried longan-like aroma, fruity aroma, and pine-smoke aroma all decreased. The intensity of dried longan-like aroma decreased to 3.01 and 1.83 in LS16 and LS13, respectively; fruity aroma decreased to 2.86 and 1.75; and pine-smoke aroma decreased to 2.49 and 1.39. In contrast, aged aroma was enhanced in LS16 and LS13, with intensities of 3.93 and 4.41, respectively, and became an important aroma attribute in samples with longer natural aging times. Previous studies have reported that the typical aroma characteristics of LS are closely associated with pine-smoke aroma and dried longan-like aroma, and that the smoking process markedly affects its volatile composition (Yao et al., 2005). Consistent with these findings, the QDA results in the present study showed that dried longan-like aroma varied among LS samples with different natural aging times and was most prominent in LS19. This suggests that dried longan-like aroma may serve as an important sensory attribute for differentiating the aroma characteristics of LS samples with different natural aging times. Therefore, aroma compound analysis was further performed to clarify the material basis of dried longan-like aroma in LS and to identify its key contributors.

### 3.2 Analysis of aroma composition in LS with different natural aging times

**Fig. 2:**
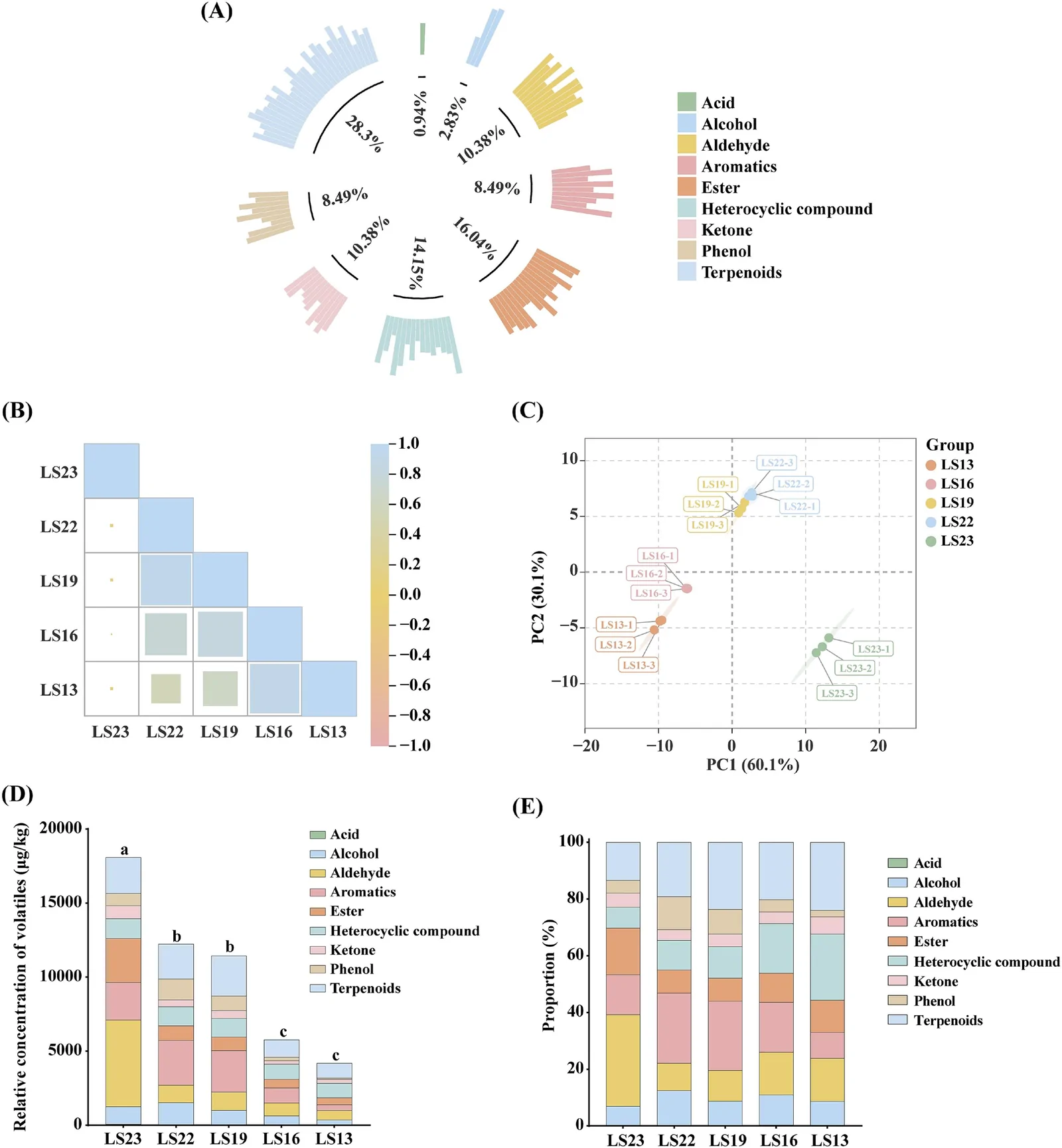
Composition and distribution of aroma components in LS from different years. (A) Classification of 106 aroma components. (B) Inter-group correlation heatmap based on aroma component contents. (C) PCA plot. (D) Stacked bar chart of the total contents of different aroma component categories in LS from different years. Different letters indicate significant differences at *P* < 0.05. (E) Stacked bar chart showing the percentage contribution of each aroma component category to the overall aroma composition of LS from different years.

To further clarify the chemical basis underlying the aroma differences among LS samples with different natural aging times, GC–MS was used to systematically characterize the volatile aroma compounds in five LS samples. A total of 106 aroma compounds were identified and semi-quantified, which were mainly classified into nine chemical categories, including 30 terpenoids (28.30%), 17 esters (16.04%), 15 heterocyclic compounds (14.15%), 11 ketones (10.38%), 11 aldehydes (10.38%), 9 phenols (8.49%), 9 aromatic compounds (8.49%), 3 alcohols (2.83%), and 1 acid (0.94%) **(**Fig. 2A **and Supplementary Table S2)**. To compare the overall differences in aroma composition among LS samples with different natural aging times, Pearson correlation analysis and hierarchical clustering heatmaps were used to evaluate the distribution patterns of volatile compounds. The correlation coefficients among samples ranged from −0.03 to 0.92. Overall, higher correlations were observed between samples with similar natural aging times, whereas lower correlations were found between samples with shorter and longer natural aging times. These results indicated that the distribution patterns of aroma compound contents changed progressively with increasing natural aging time and showed a certain degree of stage-specific differentiation (Fig. 2B). Meanwhile, the correlation coefficients among replicates within each group were close to 1, indicating good experimental reproducibility. Principal component analysis (PCA) further showed a clear separation among LS samples with different natural aging times based on their aroma composition, with PC1 and PC2 jointly explaining 90.2% of the total variance (Fig. 2C). This result further suggested, from a multivariate perspective, that LS samples with different natural aging times differed markedly in aroma composition.

The total contents and relative proportions of different chemical classes were further analyzed. As shown in Fig. 2D, the total content of aroma compounds in LS samples showed an overall decreasing trend with increasing natural aging time. This change may be associated with slow oxidation, volatilization loss, precursor transformation, and microbially related enzymatic reactions during storage (Wang et al., 2024). In addition, the relative proportions of the nine chemical classes varied markedly among LS samples with different natural aging times (Fig. 2E). Aldehydes accounted for the highest proportion in LS23, reaching 32.33%. Aromatic compounds were the dominant class in LS22 and LS19, accounting for 24.71% and 24.46%, respectively. In contrast, terpenoids represented the highest proportion in LS16 and LS13, accounting for 20.25% and 24.01%, respectively. These results indicated that increasing natural aging time was associated not only with changes in the total abundance of aroma compounds in LS, but also with a reshaping of its chemical class composition. Combined with the sensory evaluation results, the high proportion of aldehydes in LS23 may be related to its pronounced sweet aroma and fruity aroma characteristics (Zhou et al., 2023). The increased proportion of aromatic compounds in LS22 and LS19 suggested that this class of compounds may contribute to pine-smoke aroma, woody aroma, and dried longan-like aroma (Yao et al., 2005). In LS16 and LS13, the increased relative proportion of terpenoids may be related to the oxidation, volatilization, or transformation of some highly volatile compounds during storage, resulting in their relative enrichment (Tao et al., 2022). Overall, the changes in both the total content and chemical class composition of aroma compounds provided a chemical basis for the sensory transition of LS from being dominated by pine-smoke aroma, sweet aroma, and fruity aroma to a profile characterized by prominent dried longan-like aroma, followed by enhanced aged aroma and woody aroma.

### 3.3 Screening and validation of key aroma contributors to dried longan-like aroma

**Fig. 3:**
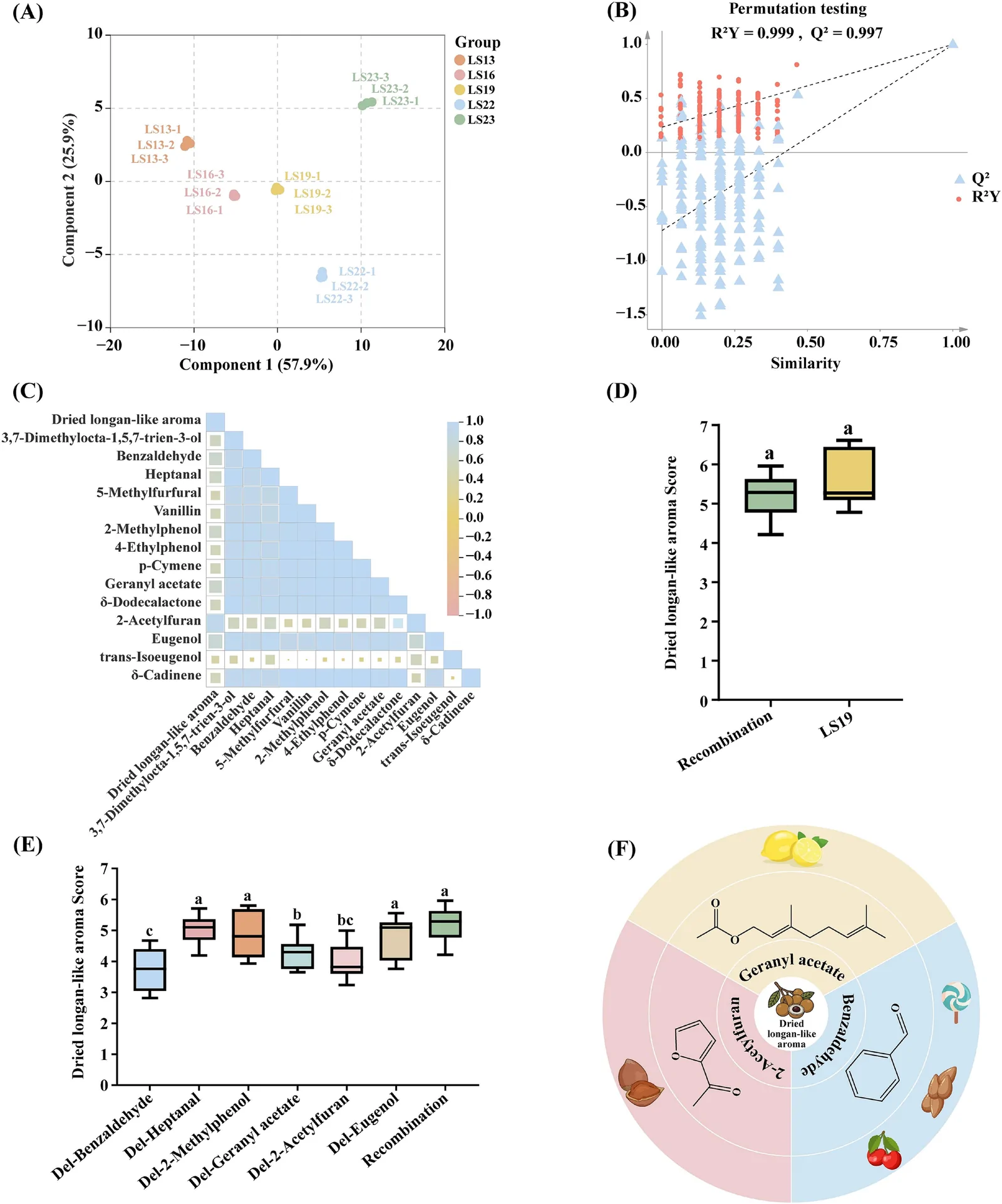
Analysis of key aroma compounds contributing to dried longan-like aroma in LS. (A) OPLS-DA plot. (B) Cross-validation of the OPLS-DA model. (C) Correlation heatmap of key aroma compounds associated with dried longan-like aroma. (D) Boxplot of the aroma recombination experiment. Different letters indicate significant differences at *P* < 0.05. (E) Boxplot of the aroma omission experiment. Different letters indicate significant differences at *P* < 0.05. (F) Aroma wheel of key aroma compounds contributing to dried longan-like aroma.

To further elucidate the key aroma compounds contributing to dried longan-like aroma in LS, an OPLS-DA model was constructed to perform multivariate discriminant analysis of aroma compounds in LS samples with different natural aging times. The results showed that the samples with different natural aging times were well separated based on their aroma compound profiles, with Component 1 and Component 2 explaining 57.9% and 25.9% of the variation, respectively (Fig. 3A). Cross-validation indicated that no obvious overfitting occurred, suggesting that the model exhibited good stability and reliability (Fig. 3B). Based on the criteria of VIP > 1.0 and *P* < 0.05, a total of 57 differential aroma compounds were screened (**Supplementary Table S3**). To further evaluate the potential aroma contributions of these differential compounds, the rOAVs were calculated. The rOAV integrates the relative content of an aroma compound and its odor threshold and can therefore be used as a preliminary indicator of its potential contribution to the overall aroma profile. Compounds with rOAV >1 are generally considered to make important contributions to sample aroma, and higher rOAV values indicate greater potential aroma impact (Huang et al., 2026). A total of 14 compounds showed rOAV values greater than 1, including four aldehydes, three aromatic compounds, two esters, two phenols, one alcohol, one heterocyclic compound, and one terpenoid (**Supplementary Table S4**). On this basis, correlations between the aroma-active compounds with rOAV >1 and the intensity of dried longan-like aroma were further analyzed. The results showed that dried longan-like aroma was significantly and positively correlated with Benzaldehyde (*r* = 0.69), Heptanal (*r* = 0.64), 2-Methylphenol *(r* = 0.66), Geranyl acetate (*r* = 0.68), 2-Acetylfuran (*r* = 0.95), and Eugenol (*r* = 0.76) (Fig. 3C and **Supplementary Table S5**). Based on the differential analysis, rOAV results, and correlation analysis, Benzaldehyde, Heptanal, 2-Methylphenol, Geranyl acetate, 2-Acetylfuran, and Eugenol were preliminarily identified as candidate key aroma compounds associated with dried longan-like aroma in LS.

To verify the actual sensory contributions of these candidate compounds to dried longan-like aroma, aroma recombination and omission experiments were further performed. An aroma recombination model was constructed by adding the six candidate aroma compounds to a deodorized tea powder matrix according to their measured concentrations in LS19, and the model was then compared with the original LS19 sample. No significant difference in dried longan-like aroma intensity was observed between the recombination model and LS19 (Fig. 3D). Although some differences in other sensory attributes were still detected, the overall aroma description of the recombination model was close to the dried longan-like aroma characteristic of LS19. This result indicated that the six aroma-active compounds could effectively reconstruct the main dried longan-like aroma profile of LS19. The contribution of each individual compound was further evaluated using omission experiments. Among the six omission models, the omission of Benzaldehyde, Geranyl acetate, or 2-Acetylfuran resulted in a significant decrease in dried longan-like aroma intensity (Fig. 3E), indicating that these three compounds were key contributors to this aroma attribute in LS. In contrast, omission of Heptanal, 2-Methylphenol, or Eugenol did not cause a significant change in dried longan-like aroma intensity, suggesting that their individual contributions were relatively weak or that their effects may be influenced by masking or interactions among compounds. Taken together, Benzaldehyde, Geranyl acetate, and 2-Acetylfuran were identified as the key aroma contributors to dried longan-like aroma in LS. To further visualize the sensory contributions of the key aroma compounds, an aroma wheel was constructed (Fig. 3F).

### 3.4 Perceptual interactions among key aroma contributors to dried longan-like aroma

**Fig. 4:**
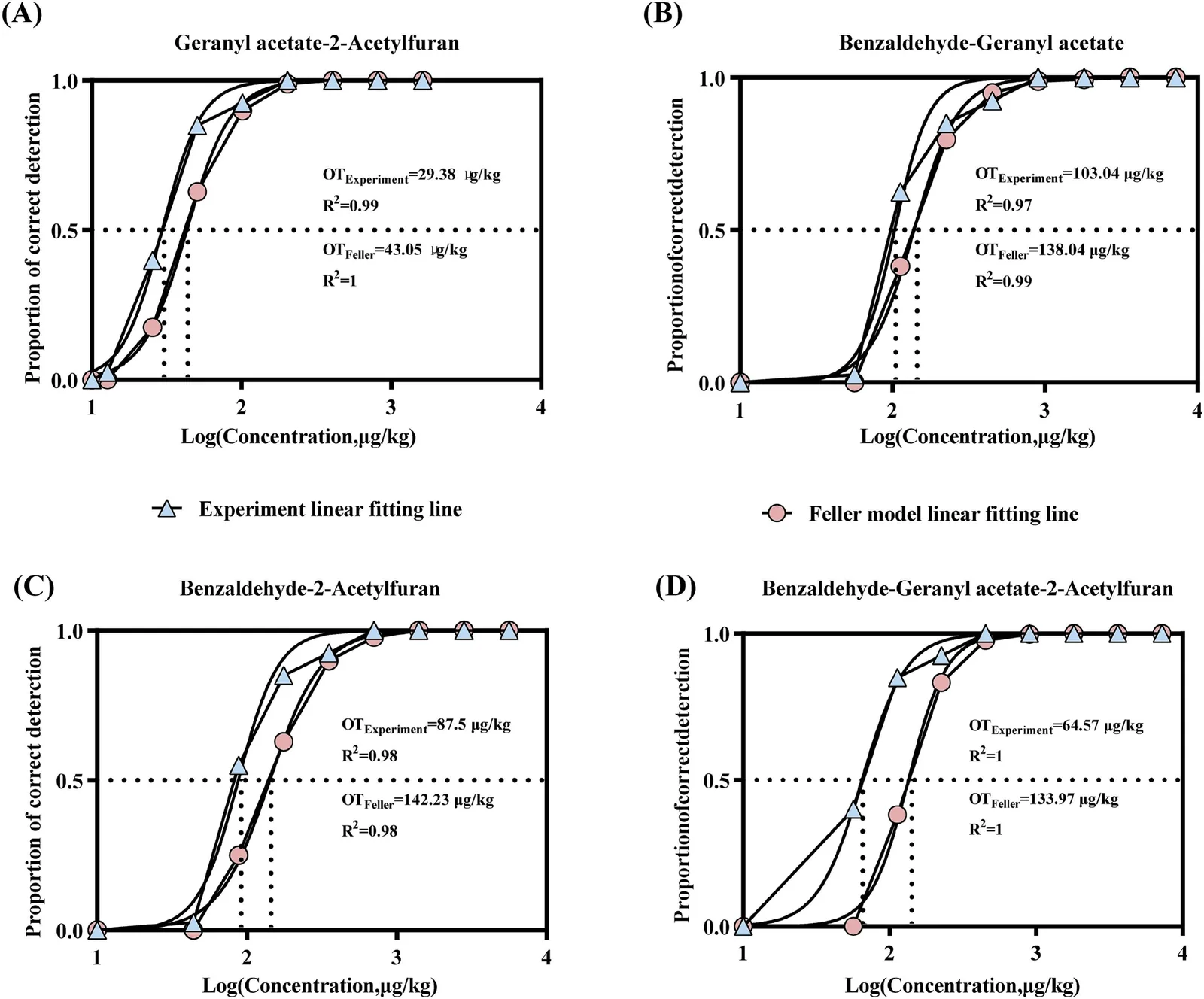
Analysis of interaction effects among key aroma compounds contributing to dried longan-like aroma. (A) Interaction effect of the binary mixture of Geranyl acetate and 2-Acetylfuran. (B) Interaction effect of the binary mixture of Benzaldehyde and Geranyl acetate. (C) Interaction effect of the binary mixture of Benzaldehyde and 2-Acetylfuran. (D) Interaction effect of the ternary mixture of Benzaldehyde, Geranyl acetate, and 2-Acetylfuran.

To further clarify the perceptual interactions among the key aroma contributors to dried longan-like aroma, the experimentally determined thresholds of different aroma combinations were compared with the theoretical thresholds calculated using the Feller additive model. The experimental-to-theoretical threshold ratios of the three binary combinations and one ternary combination ranged from 0.48 to 0.75 (**Supplementary Table S6**). Among them, the three binary combinations mainly showed additive effects, whereas the ternary combination exhibited a synergistic effect. Specifically, the experimental thresholds of Benzaldehyde–Geranyl acetate, Benzaldehyde–2-Acetylfuran, and Geranyl acetate–2-Acetylfuran were 103.04, 87.50, and 29.38 μg/kg, respectively, all of which were lower than their corresponding theoretical thresholds. The experimental-to-theoretical threshold ratios were 0.75, 0.62, and 0.68, respectively, indicating that certain mutual enhancement occurred between the components in the binary systems, although the overall interaction type was still classified as additive (Fig. 4A–C). Among these binary mixtures, Benzaldehyde–2-Acetylfuran showed the lowest ratio, suggesting that the interaction between Benzaldehyde and 2-Acetylfuran was relatively stronger and may constitute an important aroma basis for dried longan-like aroma. Compared with the binary combinations, the synergistic effect of the Benzaldehyde–Geranyl acetate–2-Acetylfuran ternary system was more pronounced. Its experimental threshold was 64.57 μg/kg, which was markedly lower than the theoretical threshold of 133.97 μg/kg, and the experimental-to-theoretical threshold ratio decreased to 0.48 (Fig. 4D). These results indicated that the coexistence of the three key aroma compounds further reduced the perceptual threshold of the mixed system and enhanced the overall perception of dried longan-like aroma. Therefore, dried longan-like aroma in LS was inferred to be a complex aroma attribute jointly shaped by Benzaldehyde, Geranyl acetate, and 2-Acetylfuran at specific concentration ratios, rather than a linear contribution from a single compound.

**Fig. 5:**
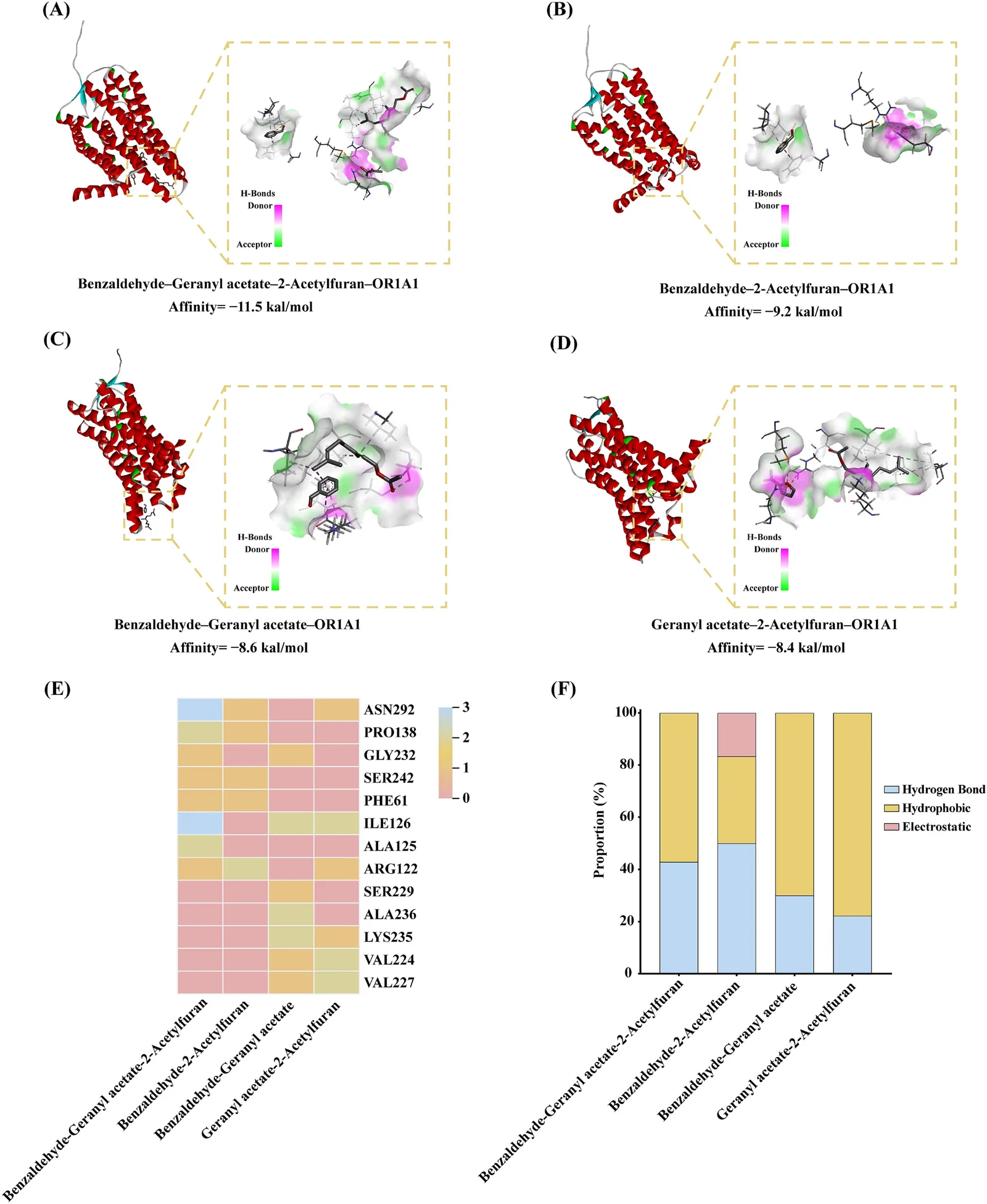
Molecular docking analysis. (A) Interaction diagram of the Benzaldehyde–Geranyl acetate–2-Acetylfuran–OR1A1 complex. (B) Interaction diagram of the Benzaldehyde–2-Acetylfuran–OR1A1 complex. (C) Interaction diagram of the Benzaldehyde–Geranyl acetate–OR1A1 complex. (D) Interaction diagram of the Geranyl acetate–2-Acetylfuran–OR1A1 complex. (E) Heatmap showing the number of interacting residues. (F) Stacked bar chart showing the proportions of interaction types.

Molecular docking can be used to predict the binding modes between flavor molecules and receptor proteins, thereby providing molecular-level evidence for the potential receptor recognition of aroma compounds (Sun et al., 2023). OR1A1 is a typical G protein-coupled receptor involved in odorant recognition and olfactory signal transduction (Liu et al., 2025). Previous studies have shown that OR1A1 exhibits a relatively broad odorant response profile, and its ligand recognition properties have been used in studies of olfactory receptor recognition mechanisms (Zhao et al., 2024). Therefore, OR1A1 and OR2W3 were selected as candidate olfactory receptors to evaluate the potential interactions between the three key aroma contributors and these receptors. As shown in Supplementary Table S7, the Benzaldehyde–Geranyl acetate–2-Acetylfuran system exhibited the most favorable binding affinity toward both OR1A1 and OR2W3, with binding energies of −11.5 and −10.7 kcal/mol, respectively. When Benzaldehyde was omitted, the binding energy changed to −8.4 kcal/mol, indicating that Benzaldehyde contributed to the binding stability of the multi-component system. This trend in binding energy was generally consistent with the sensory evaluation results, indicating that Benzaldehyde, Geranyl acetate, and 2-Acetylfuran may jointly contribute to the enhanced perception of dried longan-like aroma by stabilizing the receptor-binding conformation. Based on the relatively favorable binding performance of the ternary aroma system with OR1A1, further interaction analysis was focused on the OR1A1-ligand complexes. With respect to the binding mode, Benzaldehyde–Geranyl acetate–2-Acetylfuran formed six hydrogen bonds with ASN292, PRO138, GLY232, and SER242 in the binding pocket of OR1A1, with hydrogen bond distances ranging from 2.21 to 3.09 Å. Multiple hydrophobic interactions were also formed with PHE61, ILE126, ALA125, ARG122, and PRO138, with interaction distances ranging from 3.50 to 4.83 Å (Fig. 5A). The Benzaldehyde–2-Acetylfuran system formed three hydrogen bonds with ARG122, ASN292, and SER242, hydrophobic interactions with PHE61 and PRO138, and an electrostatic interaction with ARG122 (Fig. 5B). The Benzaldehyde–Geranyl acetate system formed three hydrogen bonds with SER229, GLY232, and ALA236, together with hydrophobic interactions involving LYS235, VAL224, VAL227, ILE126, and ALA236 (Fig. 5C). The Geranyl acetate–2-Acetylfuran system formed two hydrogen bonds with ARG122 and ASN292, and hydrophobic interactions with ILE126, LYS235, VAL224, and VAL227 (Fig. 5D). These results indicated that the three key aroma compounds could form a multi-point interaction network within the OR1A1 binding pocket through hydrogen bonds, hydrophobic interactions, and electrostatic interactions. In particular, the ternary complex exhibited more diverse binding sites and interaction types. To further identify potential key binding sites, residues with binding frequencies greater than 2 were defined as high-frequency binding residues and are highlighted in Fig. 5E. ASN292, ILE126, ARG122, LYS235, VAL224, and VAL227 were identified as the main high-frequency binding residues, suggesting that the key aroma compounds associated with dried longan-like aroma may interact with OR1A1 mainly through these sites. The statistics of interaction types showed that hydrophobic interactions accounted for 57.14%, 33.33%, 70.00%, and 77.78% of the total interactions in the four complexes, respectively, whereas hydrogen bonds accounted for 42.86%, 50.00%, 30.00%, and 22.22%, respectively (Fig. 5F). In addition, electrostatic interactions were observed in the Benzaldehyde–2-Acetylfuran–OR1A1 complex, accounting for 16.67%. Overall, hydrophobic interactions appeared to be the major force maintaining the stability of most receptor–ligand complexes, while hydrogen bonds and electrostatic interactions further contributed to receptor–ligand stabilization. These findings provide a potential molecular explanation, from the perspective of candidate olfactory receptor binding, for the involvement of Benzaldehyde, Geranyl acetate, and 2-Acetylfuran in the enhanced perception of dried longan-like aroma.

### 3.5 Molecular dynamics simulation

**Fig. 6:**
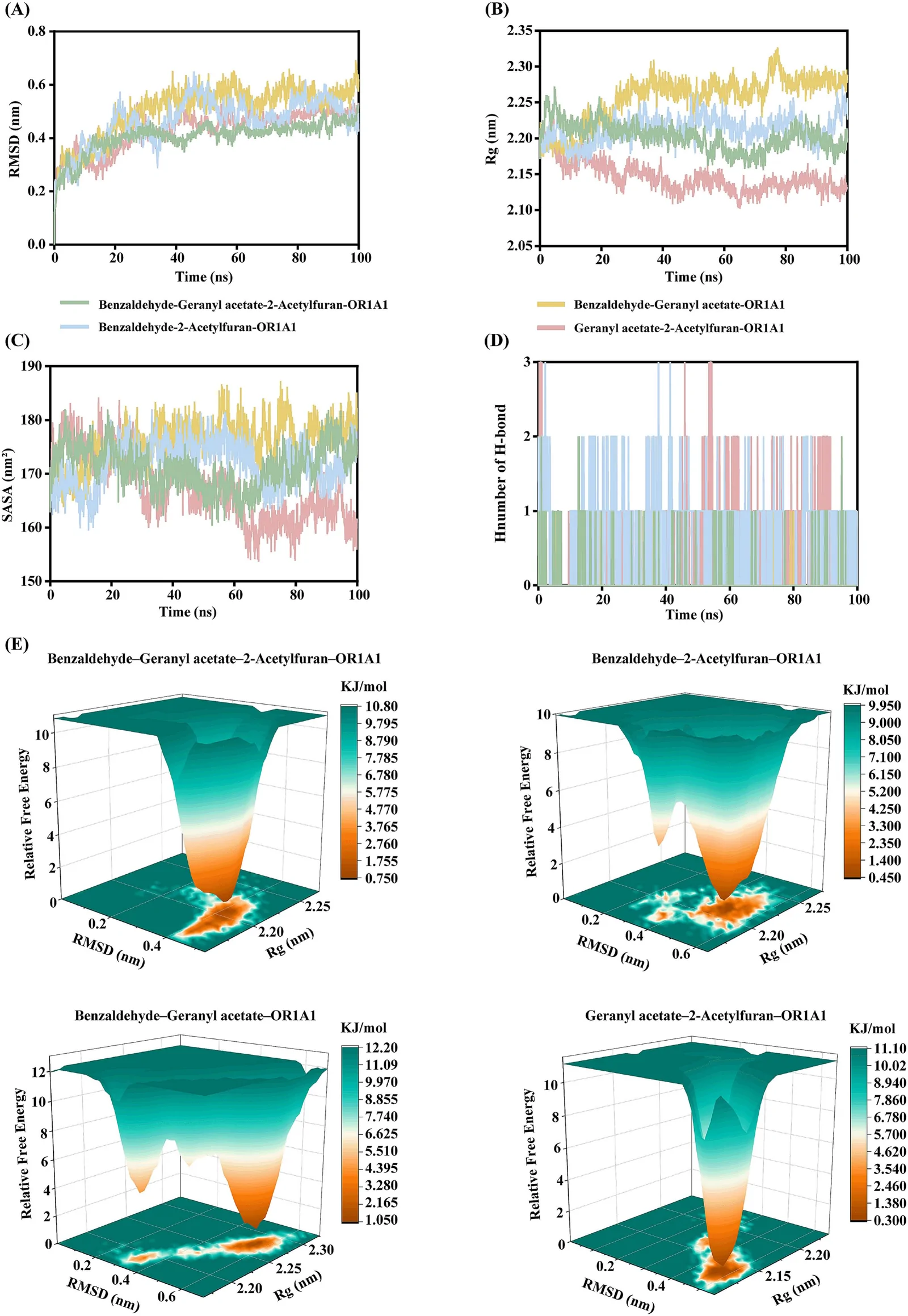
Molecular dynamics simulation analysis of the four complexes. (A) RMSD of the four complexes. (B) Rg of the four complexes. (C) SASA of the four complexes. (D) Number of hydrogen bonds in the four complexes. (E) Free energy landscapes of the four complexes.

To further evaluate the binding stability of the four complexes, MD simulations were performed based on the docking conformations. RMSD is an important parameter for characterizing the overall structural fluctuation of a complex, and its trajectory can be used to assess system stability during simulation (Xiao et al., 2024a). As shown in Fig. 6A, the RMSD values of the four complexes were 0.41 ± 0.06, 0.46 ± 0.09, 0.51 ± 0.09, and 0.43 ± 0.07 Å, respectively. Only small fluctuations were observed, and the trajectories tended to reach a plateau during the later stage of simulation, indicating that relatively stable equilibrium conformations were achieved for all complexes. The Rg was further analyzed to evaluate the compactness of the protein conformation (Xiao et al., 2024b). The Rg values of the four complexes were 2.20 ± 0.02, 2.21 ± 0.02, 2.26 ± 0.03, and 2.15 ± 0.02 nm, respectively, with only minor fluctuations throughout the simulations. These results suggested that the overall conformation of OR1A1 remained relatively compact after ligand binding (Fig. 6B). SASA was used to reflect the degree of solvent exposure and conformational unfolding of the protein (Wu et al., 2025). The SASA values of the four complexes were 171.23 ± 3.90, 171.99 ± 4.05, 176.00 ± 4.03, and 167.23 ± 6.17 nm^2^, respectively, and no obvious conformational unfolding was observed during the simulations (Fig. 6C), further indicating good structural stability of these systems in the aqueous environment. Hydrogen bond analysis showed that a relatively low number of hydrogen bonds was maintained in all four complexes during the simulations (Fig. 6D), suggesting that hydrogen bonding was not the only force responsible for maintaining stable complex conformations. Combined with the molecular docking results, hydrophobic interactions may play a more dominant role in stabilizing these complexes. Free energy landscape analysis was further conducted to reveal the energy distribution of the complexes in conformational space. As shown in Fig. 6E, relatively concentrated low-free-energy basins were observed for all four complexes, corresponding to the convergent regions of RMSD and Rg. The Benzaldehyde–Geranyl acetate–2-Acetylfuran–OR1A1 complex reached its lowest-free-energy conformation at an RMSD of 0.41 nm and an Rg of 2.21 nm. The Benzaldehyde–2-Acetylfuran–OR1A1 complex showed its lowest-free-energy conformation at an RMSD of 0.48 nm and an Rg of 2.22 nm. The Benzaldehyde–Geranyl acetate–OR1A1 complex reached its lowest-free-energy conformation at an RMSD of 0.56 nm and an Rg of 2.28 nm, while the Geranyl acetate–2-Acetylfuran–OR1A1 complex reached its minimum at an RMSD of 0.46 nm and an Rg of 2.13 nm. Overall, stable binding conformations were maintained in all four complexes. Notably, the low-free-energy region of the Benzaldehyde–Geranyl acetate–2-Acetylfuran–OR1A1 complex was more concentrated, suggesting that the ternary complex exhibited superior conformational stability. This result provided dynamic support for the potential cooperative contribution of the three key aroma compounds to the perception of dried longan-like aroma.

To further elucidate the energetic basis of the interactions in the four complexes, binding free energies were calculated using the MM/PBSA and MM/GBSA methods. Binding free energy is mainly determined by van der Waals energy, electrostatic energy, polar solvation energy, and nonpolar solvation energy. As shown in **Supplementary Table S8**, the van der Waals energies of the four complexes were − 57.93 ± 0.03, −40.05 ± 0.24, −48.05 ± 0.07, and − 46.36 ± 0.35 kcal/mol, respectively, all of which were negative, indicating that van der Waals interactions favored complex stabilization. Among them, the Benzaldehyde–Geranyl acetate–2-Acetylfuran–OR1A1 complex exhibited the lowest van der Waals energy, suggesting stronger hydrophobic interactions and better spatial complementarity. The electrostatic energies of the four complexes were − 3.39 ± 0.22, −13.33 ± 0.85, −0.98 ± 0.02, and − 8.54 ± 0.10 kcal/mol, respectively, indicating that electrostatic interactions also contributed to complex binding. The overall binding free energy results showed that the Benzaldehyde–Geranyl acetate–2-Acetylfuran–OR1A1 complex had the lowest total binding free energy, at −53.89 ± 0.27 kcal/mol, which was lower than those of the three binary complexes. This trend was consistent with the molecular docking results, in which the ternary complex also showed the lowest binding energy. These findings further indicated that the coexistence of Benzaldehyde, Geranyl acetate, and 2-Acetylfuran could enhance their binding stability with OR1A1, providing thermodynamic support for a cooperative stabilizing effect among the three compounds.

To identify key residues involved in the binding of the dried longan-like aroma contributors to OR1A1, alanine scanning analysis was performed on the Benzaldehyde–Geranyl acetate–2-Acetylfuran–OR1A1 complex. As shown in **Supplementary Table S9**, mutation of ILE126 and VAL227 to alanine increased the binding free energy by 1.09 and 1.08 kcal/mol, respectively, indicating that these two residues made important contributions to the binding affinity and conformational stability of the ternary complex. Taken together, the MD simulation, binding free energy calculation, and alanine scanning results demonstrated that Benzaldehyde, Geranyl acetate, and 2-Acetylfuran could form a stable multi-point interaction network with OR1A1 through key residues such as ILE126 and VAL227. Van der Waals interactions were identified as the main driving force for maintaining the stability of the ternary complex, while electrostatic interactions and hydrogen bonds also contributed to the stabilization of the binding conformation. These results provide a potential molecular explanation, from the perspective of receptor-binding stability and energetic contribution, for the involvement of the three key aroma compounds in the enhanced perception of dried longan-like aroma.

## 4 Conclusion

In this study, sensory evaluation, HS-SPME-GC–MS, aroma recombination and omission experiments, S-curve–Feller additive model analysis, and molecular simulation were integrated to systematically investigate the changes in aroma characteristics, the key contributors to dried longan-like aroma, and its complex perceptual basis in LS samples with different natural aging times. The results showed that the aroma characteristics of LS differed markedly among different natural aging times. With increasing natural aging time, the aroma profile of LS shifted from being dominated by pine-smoke aroma, sweet aroma, and fruity aroma to a profile characterized by prominent dried longan-like aroma, followed by enhanced woody aroma and aged aroma. Based on volatile compound analysis, rOAV calculation, correlation analysis, and sensory validation, Benzaldehyde, Geranyl acetate, and 2-Acetylfuran were identified as the key contributors to dried longan-like aroma in LS. Further perceptual interaction analysis indicated that the contributions of these three compounds were not simply linearly additive. The binary mixtures mainly showed additive effects, whereas the ternary system exhibited a stronger promoting effect, suggesting that dried longan-like aroma was a complex perceptual attribute jointly shaped by Benzaldehyde, Geranyl acetate, and 2-Acetylfuran under specific concentration relationships. Molecular docking and molecular dynamics simulations showed that the three key contributors could form relatively stable multi-point interactions within the binding pocket of the candidate olfactory receptor OR1A1. Hydrophobic interactions appeared to be the major force maintaining complex stability, while ILE126 and VAL227 were identified as key binding residues in OR1A1. Overall, this study clarified the natural aging-related evolution of aroma characteristics in LS and the material basis and perceptual features of dried longan-like aroma. These findings provide a candidate chemical basis and theoretical reference for LS aroma quality evaluation, storage management, and targeted regulation of characteristic aroma. Nevertheless, some limitations should be acknowledged. The present study was conducted using LS samples from a single tea-producing region under consistent storage conditions, and the molecular simulation results mainly provided computational evidence for potential aroma–receptor interactions. Future studies involving samples from multiple production regions, long-term monitoring of storage environmental parameters, and experimental validation of aroma–receptor interactions are needed to further elucidate the mechanisms underlying the formation and perception of dried longan-like aroma.

## CRediT authorship contribution statement

**Yutao Shi:** Writing – original draft, Visualization, Methodology, Investigation, Formal analysis, Data curation, Conceptualization. **Lirong Zuo:** Visualization, Investigation, Data curation. **Canyang Huang:** Investigation, Data curation. **Yanhua Zhou:** Validation, Investigation. **Xi Cheng:** Methodology, Investigation. **Yan Huang:** Investigation, Formal analysis. **Xiaoyong Wang:** Validation, Methodology, Formal analysis. **Yucheng Zheng:** Validation, Investigation, Formal analysis. **Feiquan Wang:** Resources, Investigation. **Bo Zhang:** Supervision, Project administration, Conceptualization. **Shulin Zheng:** Writing – review & editing, Supervision, Project administration, Investigation, Funding acquisition, Conceptualization.

## Ethics declaration

### Informed consent and patient details

Written informed consent to take part in the study and to publish the article has been obtained from all participants or their legal representatives. The privacy rights of participants have been observed.

### Studies in human

This study was performed in compliance with relevant laws, regulatory frameworks and guidelines where the research took place.

This study was conducted in accordance with Declaration of Helsinki and Wuyi University ethical guidelines for research involving human participants.

This study was approved by the Academic Ethics Committee of Wuyi University. (Approval No. 2026021)

## Funding

This work was supported by the Nanping Science and Technology Commissioner–Oriented Industry Technology Innovation Joint Funding Program (No. N2023Z006); Science and Technology Innovation Development Fund of Wuyi University (No. 2020R1008002-2, 2020J01404-2); Special Fund for Improving the Running Level of Institutions of Higher Education in Fujian Province (Fujian Provincial Department of Finance and Department of Education [2022] No. 77); Industry-University-Research Joint Innovation Project of Fujian Province (No. 2023N5013); the Open Fund of Collaborative Innovation Center of Chinese Oolong Tea Industry (2025W01).

## Declaration of competing interest

The authors declare that they have no known competing financial interests or personal relationships that could have appeared to influence the work reported in this study.

## Data Availability

Data will be made available on request.
