# Supplementary material for: Key aroma contributors and perceptual interactions of dried longan-like aroma in lapsang souchong black tea with different natural aging times
Source: Food Chem X. 2026 Sep 11;39:104445. doi: 10.1016/j.fochx.2026.104445 (PMC13587905; doi:10.1016/j.fochx.2026.104445)
Supplement: Supplementary file 1 [file mmc1.docx]

**Supplementary Figure**

**Supplementary Figure S1.** Total ion chromatogram (TIC) of the deodorized tea powder matrix analyzed by HS-SPME–GC–MS.
